# Expression and purification of functional human glycogen synthase-1:glycogenin-1 complex in insect cells

**DOI:** 10.1016/j.pep.2014.12.007

**Published:** 2015-04

**Authors:** Roger W. Hunter, Elton Zeqiraj, Nicholas Morrice, Frank Sicheri, Kei Sakamoto

**Affiliations:** a Nestlé Institute of Health Sciences SA, EPFL Innovation Park, bâtiment G, 1015 Lausanne, Switzerland; b Lunenfeld-Tanenbaum Research Institute, Mount Sinai Hospital, 600 University Avenue, Toronto, Ontario M5G 1X5, Canada; c Beatson Institute for Cancer Research, Bearsden, Glasgow G61 1BD, UK; d Departments of Biochemistry and Molecular Genetics, University of Toronto, Toronto, Ontario M5S 1A8, Canada

**Keywords:** Energy metabolism, Glycogenesis, Glucosyltransferase, Phosphorylation

## Abstract

We report the successful expression and purification of functional human muscle glycogen synthase (GYS1) in complex with human glycogenin-1 (GN1). Stoichiometric GYS1:GN1 complex was produced by co-expression of GYS1 and GN1 using a bicistronic pFastBac™-Dual expression vector, followed by affinity purification and subsequent size-exclusion chromatography. Mass spectrometry analysis identified that GYS1 is phosphorylated at several well-characterised and uncharacterised Ser/Thr residues. Biochemical analysis, including activity ratio (in the absence relative to that in the presence of glucose-6-phosphate) measurement, covalently attached phosphate estimation as well as phosphatase treatment, revealed that recombinant GYS1 is substantially more heavily phosphorylated than would be observed in intact human or rodent muscle tissues. A large quantity of highly-pure stoichiometric GYS1:GN1 complex will be useful to study its structural and biochemical properties in the future, which would reveal mechanistic insights into its functional role in glycogen biosynthesis.

## Highlights

• GYS1:GN1 complex expressed using bicistronic pFastBac-Dual vector in insect cells.
• A large quantity of highly-pure stoichiometric GYS1:GN1 complex obtained.
• Purified GYS1 is functional and heavily phosphorylated at several Ser/Thr residues.
• GYS1:GN1 complex will be useful to reveal its structural and biochemical properties.

## Introduction

Glycogen is a branched polymer of glucose that serves as a repository of rapidly accessible energy and, thus plays an important role in maintaining glucose and energy homeostasis at both cellular and organism levels [1]. In mammals, liver and muscle tissues are the major depots for glycogen storage, although to a much lesser extent most tissues contain non-negligible levels of glycogen. While the physiological importance of glycogen in muscle and liver has been extensively documented in the context of glucose homeostasis, recent evidence suggests a key role of glycogen in brain (in both astrocytes and neurons) for cognitive function [2] and also cancer cells for senescence and cellular growth [3].

Bulk biosynthesis of glycogen is mediated by glycogen synthase which catalyses the addition of α-1,4-linked glucose units from uridine diphosphate (UDP)^3^-glucose to a nascent glycogen chain, and branching enzyme which forms α-1,6-glycosidic branchpoints [1]. In mammals, there are two glycogen synthase isoforms: muscle glycogen synthase (GYS1; encoded by *GYS1*), which is ubiquitously expressed, but most abundantly expressed in skeletal and cardiac muscles, and the liver-specific isoform (GYS2; encoded by *GYS2*). Activity of both isoforms is regulated by a complex interplay between reversible phosphorylation at multiple sites and allosteric regulators of which glucose-6-phosphate (G6P), an activator, plays the most important role [1,4,5].

Besides glycogen synthase and branching enzyme, glycogenin is another key enzyme involved in biosynthesis of glycogen. Glycogenin (GN) initiates glycogen synthesis by generating a short linear (α-1,4-linked) chain of approximately 10 glucose units attached to a conserved tyrosine residue (i.e., Y194 in human) [1,6]. Cohen and colleagues first reported that glycogen synthase purified from rabbit skeletal muscle exists as a stoichiometric complex with glycogenin [7] and proposed a key interplay between the two enzymes in initiation of glycogen synthesis [6]. Subsequent studies have demonstrated that glycogenin interacts with glycogen synthase and enhances soluble overexpression of glycogen synthase (when ectopically co-expressed with glycogen synthase in cultured cell lines) [4,8]. This has led Khanna et al. [9] to make an attempt to co-express GYS1 and glycogenin using MultiBac system in insect cells, which resulted in a successful soluble expression and purification of large quantities of human GYS1 proteins. Direct interaction of the C-terminus of GN with glycogen synthase was first reported by Skurat et al. [10] based on a yeast two-hybrid study followed by cellular overexpression experiments and more recently by an X-ray crystal structure of glycogen synthase in complex with a C-terminal 34-residue fragment of GN [11].

Given the implication that the glycogen synthase:glycogenin complex plays an important role in the initiation and elongation of glycogen particles [6,11], it would be important to further explore their molecular interaction and function as a complete full-length complex for X-ray crystallographic and biochemical property/functional studies. We here report a successful large-scale expression and purification of human GYS1 and human GN1 in insect cells and also show biochemical properties of the enzyme complex in cell-free assay systems.

## Materials and methods

### Materials

Rabbit liver glycogen and pancreatic α-amylase were from Sigma. Microcystin-LR was from Enzo Life Sciences. NADH was from Apollo Scientific. Antibodies against pS8 GYS1 and pS8/S11 GYS1 were kindly donated by D. Grahame Hardie (University of Dundee). pS641 GYS1 (#3891) and total GYS1 (#3893) antibodies were from Cell Signaling Technologies. Total GYS1 (sc-81173) and PP1c (sc-7182) antibodies were from Santa Cruz Biotechnology. pS641/645 GYS1 (S486A, 3rd bleed) and GN1 (S197C, 1st bleed) antibodies were generated by the Division of Signal Transduction Therapy (DSTT) at the University of Dundee as previously described [4]. Peroxidase conjugated secondary antibodies were from Jackson Immunoresearch. Glutathione Sepharose 4B, NHS-Sepharose FF, Superdex 200 3.2/300, Q-Sepharose HP and Enhanced Chemiluminescent (ECL) reagent were from GE Healthcare. Gel filtration standards were from Bio-Rad (151-1901). All other reagents, unless otherwise indicated, were from Sigma.

### Expression and purification of GYS1:GN1 complex

Genes coding for human GYS1 (NM 002103) and GST-tagged GN1 (NM 004130) were cloned in a pFastBac™-Dual vector (Life Technologies). A PreScission protease site was added between GST and GN1. Recombinant bacmid was generated in DH10Bac™ cells (Life Technologies) and extracted using phenol:chloroform. Unless specified otherwise, *Spodoptera* *frugiperda* (Sf9) cells (Life Technologies) were cultured in suspension flasks on a rotating platform (110 RPM at 26 °C) using an incubator shaker (Infors HT) and Sf-900 II media following the supplier’s manual. Recombinant progeny 1 (P1) baculovirus was produced in monolayer cultures by transfecting 5 ml Sf9 cells (1.0 × 10^6^ cells/ml grown in 25 cm^2^ flasks) and was harvested after 6 days, aliquoted and stored at −80 °C. P2 virus was generated by infecting Sf9 suspension cultures (1.5 × 10^6^ cells/ml) with P1 virus (400 μl/100 ml cells). The supernatant from this culture (P2) was harvested 72 h post-infection, and 3 ml were used to infect 600 ml of Sf9 suspension culture (1.5 × 10^6^ cells/ml), thus generating a P3 virus 72 h post-infection. This culture (P3) was used at a 1:10 ratio to infect 2 L of Sf9 cells (1.5–2.0 × 10^6^ cells/ml) for protein production. Cells were grown in suspension for 48 h and the cell pellets were washed in PBS prior to harvesting in ice-cold lysis buffer (50 mM tris–HCl pH 7.8, 150 mM NaCl, 5% (v/v) glycerol, 1 mM EDTA, 1 mM EGTA, 1 mM benzamidine, 0.2 mM PMSF, 5 μM leupeptin and 0.1% (v/v) 2-mercaptoethanol). Cells were lysed using a continuous flow cell disruptor at 18,000 psi and the lysate was clarified by centrifugation at 26,000*g* for 30 min. The supernatant was incubated on a rolling platform for 1 h at 4 °C with 1 ml of glutathione–Sepharose resin, pre-equilibrated in low salt buffer (50 mM tris–HCl pH 7.8, 150 mM NaCl, 5% (v/v) glycerol, 1 mM EDTA, 1 mM EGTA, 1 mM benzamidine and 0.1% (v/v) 2-mercaptoethanol). The beads were washed with 10 column volumes (CV) of low salt buffer and 50 CV of high salt buffer (low salt buffer containing 500 mM NaCl), followed by 10 CV of low salt buffer. Proteins were eluted by incubating with 0.2 mg of GST-tagged PreScission protease for 16 h at 4 °C. The eluate was concentrated to 0.7–1 ml and loaded on a Superdex 200 10/300 column (GE Healthcare), pre-equilibrated in 25 mM tris–HCl pH 7.8, 150 mM NaCl and 1 mM TCEP. Eluted peaks were analysed by SDS–PAGE, and fractions containing >95% pure GYS1-GN1 complex were combined, concentrated to 2–3 mg/ml, snap frozen in liquid nitrogen and stored at −80 °C.

### Preparation of recombinant GST-PP1c

GST-tagged human PP1 catalytic subunit *γ* was cloned into pGEX6P-1 and expressed in BL21-CodonPlus (DE3)-RIL, cultured in LB media supplemented with 1 mM MnCl_2_ at 18 °C post induction with 0.2 mM IPTG. Cultures were harvested and lysed in 50 mM tris pH 7.5, 300 mM NaCl, 0.1 mM EDTA, 10% (w/v) glycerol, 1 mM MnCl_2_, 0.1% (v/v) 2-mercaptoethanol and 0.5 mM PMSF by sonication. GST-PP1c was purified sequentially using glutathione Sepharose 4B and Q-Sepharose HP and stored in 25 mM tris pH 7.5, 300 mM NaCl, 0.1 mM EDTA, 1 mM MnCl_2_, 0.1% (v/v) 2-mercaptoethanol and 50% (v/v) glycerol at −20 °C.

### Mass spectrometry analysis

GYS1:GN1 complex was separated by SDS–PAGE and stained with colloidal Coomassie staining solution. The GYS1 band was excised, destained, in-gel reduced and alkylated with iodoacetamide, then dried with acetonitrile followed by Speedvac. Samples were digested with 60 μl of 2 μg/ml trypsin in 50 mM triethylammonium bicarbonate (TEAB) pH 8 overnight. Peptides were extracted with an equal volume of acetonitrile, dried and redissolved in 60 μl 5% acetonitrile/10 mM TEAB. Samples were analysed by LC-MS on a 180 × 0.075 mm packed emitter coupled to an Orbitrap velos mass spectrometry system. Peptides were separated over a 60 min gradient and the 10 most abundant precursors detected in the Orbitrap at 60,000 resolution (2–4^+^ charge state) were selected for MS/MS in the LTQ velos. To ensure the best fragmentation spectrum for any phosphopeptide was obtained, the precursors were fragmented using multistage activation (MSA) of the precursor mass (*m*/*z*) minus 49, 32.33 and 24.25 Da (this represents the loss of H_3_PO_4_ from a 2^+^, 3^+^ or 4^+^ precursor ion). Raw files were processed using Proteome Discoverer 1.4 and searched using Mascot 2.3 against the Swissprot database (human only), allowing for carbamidomethyl modification of cysteine, oxidation of methionine and phosphorylation of Ser/Thr/Tyr.

### Phosphate content

Covalent phosphate was estimated by alkaline hydrolysis followed by determination of released phosphate by complexometry with a malachite green/molybdate reagent [12].

### Electrophoresis and Western blotting

Samples were denatured in Laemmli sample buffer, separated on 8–10% acrylamide tris–glycine gels and stained with Coomassie brilliant blue (CBB) G-250 [13] or transferred to polyvinylidene difluoride membrane in Towbin buffer for 1 h at 100 V constant. Membranes were blocked in 5% (w/v) milk prepared in tris-buffered saline containing 0.1% (v/v) Tween-20 (TBST) for 1 h at room temperature and incubated overnight in primary antibody. Membranes were washed with TBST and incubated with peroxidase conjugated secondary antibodies for 1 h at room temperature before detection with ECL reagent.

### *In vitro* dephosphorylation

GYS1:GN1 preparations were dephosphorylated with purified GST-PP1c in reactions containing 20 mM TES pH 7.4, 100 mM KCl, 5 mM MnCl_2_, 0.1 mM EGTA, 0.01% (w/v) BRIJ-35 and 0.1% (v/v) 2-mercaptoethanol for 30 min at 30 °C. Reactions were terminated by the addition of 100 nM microcystin-LR prior to assay or by denaturation in Laemmli sample buffer.

### *In vitro* deglycosylation

GYS1:GN1 preparations were incubated with 50 mU α-amylase immobilised on NHS-Sepharose in 25 mM TES pH 7.4, 100 mM NaCl and 5 mM CaCl_2_ for 30 min at 37 °C. Reactions were terminated by denaturation in Laemmli sample buffer.

### Glycogen synthase assay

Glycosyltransferase activity was determined using a coupled spectrophotometric assay to monitor the formation of UDP in reactions containing 50 mM TES pH 7.4, 100 mM KCl, 2 mM MgCl_2_, 1 mM phosphoenolpyruvate, 0.02% (w/v) bovine serum albumin, 2 mM 2-mercaptoethanol, 20 U/ml pyruvate kinase and 2 U/ml lactate dehydrogenase. Enzyme preparations were incubated in 1% (w/v) glycogen for 15 min at 30 °C prior to assay and added to reactions to yield a final concentration of 0.1% (w/v) glycogen (5.5 mM glucose equivalent.) and 1 mU GYS1. Reactions were started by the addition of UDP-glucose and absorbance at 340 nm (*A*_340_) recorded for 10 min at 30 °C. Results are expressed as U/mg (GYS1 only, not total protein), where 1 U = 1 μmol UDP formed per min at 30 °C assuming ε340 [NADH] = 6.22 mM^−1^ cm^−1^. Kinetic parameters were determined by non-linear regression using Graphpad Prism v5.02 and the following equation:

$$v=\frac{v_{\text{max}}\times {\left[S\right]}^{h}}{K_{m}^{h}+{\left[S\right]}^{h}}$$

where *v* = velocity, [*S*] = concentration of substrate or activator, *K*_m_ = concentration of substrate or activator that increases velocity to 50% maximal stimulated activity and *h* = Hill coefficient.

## Results and discussion

### Co-expression of human muscle glycogen synthase (GYS)-1 and human glycogenin (GN)-1 in a bicistronic bacmid in *S. frugiperda*

**Fig. 1:**
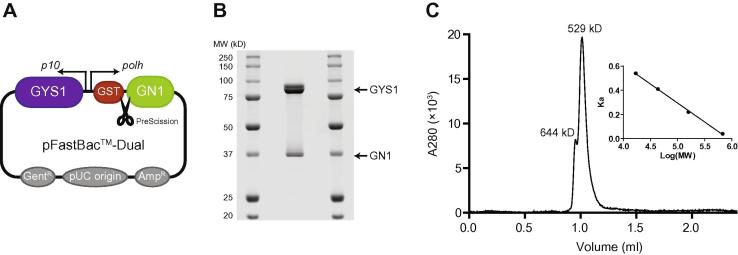
Vector design and purification of GYS1:GN1. (A) Diagram illustrating the key features of the bicistronic vector used to co-express human GYS1 and GST-tagged GN1. Arrows indicate p10 and polyhedrin (polh) promoters. Ampicillin (Amp^R^) and gentamicin (Gent^R^) resistance genes and origin of replication (pUC) are also labelled. (B) Representative image of a CBB-stained SDS–PAGE gel of the purified recombinant GYS1:GN1 complex (2 μg). The relative mobilities of GYS1 and GN1 are indicated with arrows. (C) Estimation of complex MW by size exclusion chromatography. GYS1:GN1 (5 μg) was fractionated on a Superdex 200 3.2/300 column equilibrated with 20 mM TES pH 7.4, 150 mM KCl and 1 mM DTT. Shown inset is a calibration curve of Ka [(Vt − Vo)/(Ve − Vo), where Vt = total column volume, Vo = void volume and Ve = elution volume)] vs. log_10_ MW prepared using protein standards.

Taking advantage of a bicistronic expression vector (Fig. 1A), we were able to successfully co-express human GYS1 with GST-tagged GN1 in insect *S.* *frugiperda* (Sf9) cells. GYS1 and GST-GN1 co-eluted after glutathione Sepharose affinity purification and subsequent size-exclusion chromatography. The GYS1:GN1 complex produced in this manner was >95% pure as assessed by SDS–PAGE and had a yield of ∼1 mg/L of Sf9 culture (Fig. 1B).

Interestingly, the apparent molecular weight of the GYS1:GN1 complex estimated by size-exclusion chromatography was 529 kD, suggestive of a hetero-oligomeric structure with 4:4 stoichiometry (Fig. 1C). This is similar to the size of the native GYS1:GN1 active complex isolated from rabbit muscle tissues [7] indicating the preparations are representative of a physiologically relevant form. The 4:4 stoichiometry is also consistent with previous studies showing that *Saccharomyces* *cerevisiae* and *Caenorhabditis* *elegans* GYS form a functional tetramer [11,14] and that each GYS monomer is capable of interacting with a GN1 C-terminus docking motif [11]. A GYS1:GN1 complex with molecular weight larger than a 4:4 complex was also apparent from the small shoulder peak (Fig. 1C). It is not clear if this represents a biologically relevant complex or if this is a protein aggregate that appears under *in vitro* conditions.

### GYS1:GN1 complex is stoichiometrically phosphorylated on key regulatory sites

**Fig. 2:**
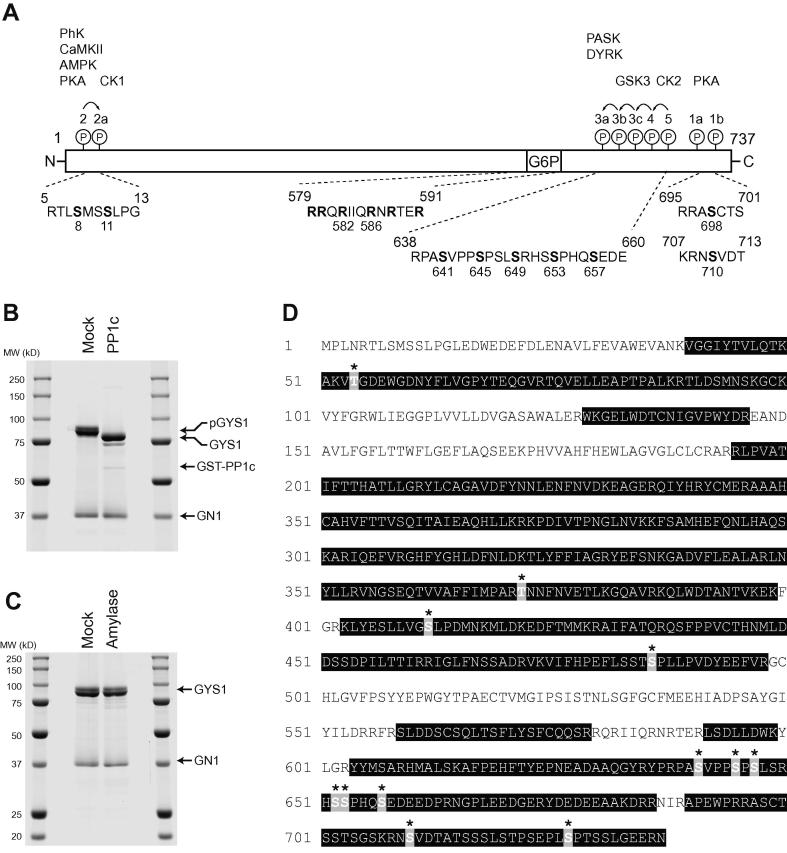
GYS1:GN1 prepared in *S. frugiperda* is heavily phosphorylated. (A) Domain organisation and known *in vivo* phosphorylation sites of human GYS1 (P13807). Phosphorylation sites are labelled with the historical nomenclature (1–5) and sequence numbering from the initiator methionine. Protein kinases that phosphorylate these sites (*in vitro* and *in vivo*) are indicated. The conserved arginine rich cluster required for G6P activation is also highlighted. (B) Dephosphorylation of GYS1:GN1 with PP1c results in a marked increase in electrophoretic mobility. GYS1:GN1 was incubated with PP1c as described in methods and fractionated by SDS–PAGE prior to staining with CBB. (C) Alternatively, GYS1:GN1 was incubated with α-amylase to remove carbohydrate. A representative CBB-stained gel is shown. (D) Post-translational modifications of GYS1 were determined by LC-MS of tryptic peptides as described in methods. The full sequence of human GYS1 is shown (P13807). Sequence coverage is highlighted by shading and mapped sites indicated with an asterisk. Key for (A): PhK – Phosphorylase kinase, CaMKII – Ca^2+^/calmodulin-dependent protein kinase, AMPK – AMP-activated protein kinase, PKA – cAMP-dependent protein kinase, CK1/2 – casein kinase 1/2, PASK – Per-Arnt-Sim domain containing serine/threonine kinase, DYRK – dual specificity tyrosine-regulated kinase, GSK3 – glycogen synthase kinase 3.

GYS is regulated by a complex interplay between positive allosteric stimulation by G6P binding and inhibition by covalent modification by reversible phosphorylation [1]. There are nine well-characterised phosphorylation sites on GYS1 clustered at both the N- and C-termini (Fig. 2A). Mutagenesis experiments have revealed that sites 2, 2a, 3a and 3b play a dominant role in regulation of GYS1 [15], whereas site 2 has the most influential role in control of GYS2 activity [16]. Interestingly, phosphorylation of these sites is also hierarchical whereby phosphorylation of site 2 by cAMP-dependent protein kinase (PKA) (or possibly by other kinases such as glycogen phosphorylase kinase and AMP-activated protein kinase [1,17,18]) enhances phosphorylation on site 2a by casein kinase 1 (CK1) [18] and CK2-dependent phosphorylation of site 5 primes the subsequent addition of four phosphates (4, 3c, 3b and 3a) by glycogen synthase kinase 3 (GSK3) [19]. In contrast, GN is a constitutively active enzyme so does not represent a point of regulation. GYS1 expressed as a complex with GN1 in insect cells was heavily phosphorylated as indicated by a significant increase in electrophoretic mobility after incubation with the catalytic subunit of protein phosphatase 1 (PP1c) (Fig. 2B). Indeed, estimation of covalently attached phosphate by alkaline hydrolysis and Malachite green reagent suggested an average stoichiometry of 6 mol:mol (phosphate:total protein), significantly higher than would be observed in intact muscle tissues. Removal of carbohydrate by α-amylase digestion had little impact on the mobility of GN apart from a subtle sharpening of the band suggesting that the degree of glycosylation was modest consistent with the highly phosphorylated, inactive state of GYS1, which would be unable to extend the primed GN1 (Fig. 2C).

The phosphorylation sites on GYS1 were mapped by tandem mass spectrometry (MS/MS) of tryptic peptides. Sequence coverage was 73% and peptides identified with a high degree of confidence are highlighted in the sequence of human GYS1 shown in Fig. 2D. Identified sites and corresponding peptides are summarised in Table 1. As expected, the highly acidic N-terminal peptide (GYS1 6–39, pI = 3.24, charge −8) containing sites 2 and 2a (S8/S11) was not detected but the remaining conventional phosphorylation sites at the C-terminus (3a, 3b, 4 and 5 or S641, S645, S653 and S657) were present on a range of multiply phosphorylated species. Indeed, many of these sites are apparently phosphorylated to near stoichiometry given the very low abundance of non-phosphorylated species. Although we were unable to formally detect S649 (3c) in the MS/MS spectra, there was clear evidence of penta, hexa and even hepta-phosphorylated forms of 641–650 so this site is likely also modified. Site 1b (S710) but not site 1a was also present, though at relatively low abundance. In addition, a number of non-conventional sites were identified (T54, T372, S412, S486, S652 and S727), albeit at relatively low abundance. Several of these sites have been identified by a recent study where human GYS1 was overexpressed in insect cells [9] and also in global MS screens [20–22]. It might be of interest to perform mutagenesis analysis and examine the impact of these phosphorylation sites on the regulation of GYS1 activity and possibly cellular localisation, as the physiological significance of these non-conventional sites is unknown.

### GYS1:GN1 complex is functional and exhibits both allosteric and phospho-dependent regulation

**Fig. 3:**
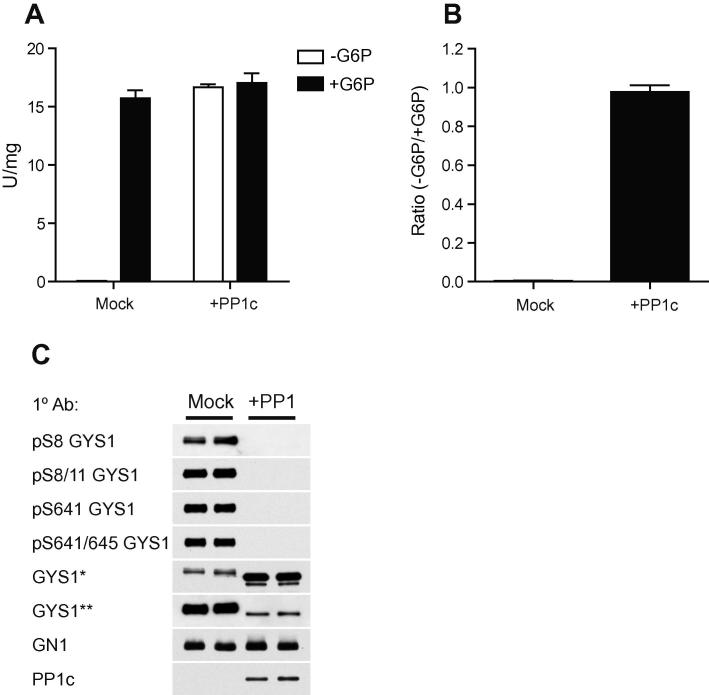
Activation of GYS1:GN1 by dephosphorylation. (A) GYS1:GN1 was incubated with buffer control (mock) or GST-PP1c as described in methods and glycosyltransferase activity determined using saturating UDP-glucose (4 mM) in the presence or absence of 10 mM G6P. Results represent mean ± SD for three independent experiments. (B) Activity ratio (−G6P/+10 mM G6P) for native (mock) and dephosphorylated (+PP1c) forms of GYS1:GN1. Results represent the mean ± SD for three independent experiments. (C) Analysis of the major regulatory phosphorylation sites on recombinant GYS1 by Western blotting of the preparations described in (A). Samples were denatured in Laemmli buffer and immunoblotted with the indicated antibodies. ^∗^Cell Signaling anti-GYS1 (#3893). ^∗∗^Santa Cruz anti-GYS1 (sc-81173, GS-7H5).

Glycosyltransferase activity of the GYS1:GN1 complex was assessed using a coupled spectrophotometric assay with pyruvate kinase/lactate dehydrogenase to follow the formation of UDP in real time. As anticipated, due to the extensive phosphorylation of GYS1 on key regulatory sites, the complex was essentially inactive under standard assay conditions (4 mM UDP-glucose) in the absence of G6P (Fig. 3A). Dephosphorylation with PP1c or the presence of saturating G6P resulted in a substantial increase in activity to ∼17 U/mg GYS1. This value is somewhat lower than reported for the classical preparation of GYS1 from rabbit skeletal muscle (∼30 U/mg), but similar to recombinant preparations of GYS isoforms prepared in various mammalian cell lines [5,17] or Hi5 cells [9]. The reason for this discrepancy is unclear, but may be due to as yet unknown post-translational modifications not replicated in heterologous expression systems. While it has been reported that GYS1 can be modified by both O-GlcNAcylation [23] and lysine acetylation [24], their functional significance is uncertain. It is not due to the common problem of partial proteolysis during isolation of GYS1 from tissue, as the N- and C-termini are known to be intact [25].

The ratio of activity in the absence and presence of G6P is commonly used as a readout of GYS phosphorylation status, albeit with certain caveats [1] and ranges between 0.1 and 0.3 in skeletal muscle depending on nutritional/hormonal status. The activity ratio (−G6P/+G6P) for native recombinant GYS1:GN1 was essentially nil, but approached unity for PP1c-treated GYS1 (Fig. 3B) indicating quantitative removal of phosphorylation on all regulatory sites which was independently confirmed by immunoblotting with site-specific antibodies (Fig. 3C). This analysis also confirmed the presence of phosphorylation at the N-terminal regulatory sites (S8/S11) which were refractory to detection by LC–MS (Table 1). Detection with the phospho-S8 antibody was relatively weak requiring significant amplification compared to phospho-S8/S11, suggesting that in most instances this region is phosphorylated in tandem. This figure also demonstrates a notably underappreciated caveat when immunoblotting GS in tissue/cell extracts, where the majority of antibodies we have tested exhibit a marked phospho-dependence (data not shown). Two examples are shown which illustrate both extremes of this phenomenon, the widely used rabbit polyclonal antibody from Cell Signaling Technologies and the GS-7H5 mouse monoclonal antibody which have a striking preference for dephospho and phospho-GYS1 respectively (equal loading is confirmed from the signal for GN1). Consequently, it is not unusual to see reported data where the apparent expression of GYS is different between nutritional states or hormone treatment, but this invariably correlates with changes in phosphorylation.

### Enzyme kinetic properties of GYS1:GN1

**Fig. 4:**
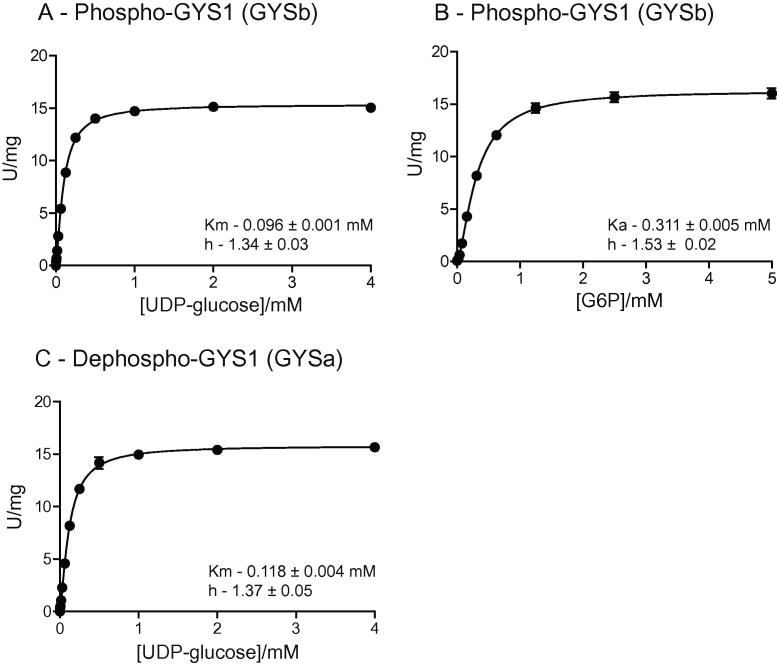
Kinetic parameters of GYS1:GN1. (A) *K*_m_(UDP-glucose) of the native, phosphorylated GYS1:GN1 complex (GYSb) was assayed as described in methods using saturating G6P (10 mM). (B) Ka(G6P) of the native GYS1:GN1 complex (GYSb) was assayed using saturating UDP-glucose (4 mM). (C) GYS1 was quantitatively dephosphorylated with PP1c and *K*_m_(UDP-glucose) determined in the absence of G6P. All results represent mean ± SD of three independent experiments.

The kinetic properties of native, phosphorylated GYS1 (GYSb) and the dephospho-form (GYSa) were determined using the coupled spectrophotometric assay. Plots of fitted data are shown in Fig 4A–C and a summary of fitted parameters is presented in Table 2. As described previously, the native form of the complex (GYSb) was effectively inactive, but could be stimulated by either dephosphorylation or the addition of G6P to essentially the same activity (∼17 U/mg). Half maximal stimulation of GYSb with G6P [Ka(G6P)] was ∼0.3 mM with a Hill coefficient (*h*) > 1 indicating a degree of positive cooperativity. The substrate activity relationship for UDP-glucose ([*K*_m_(UDPG)] for GYSa and GYSb activated with G6P was essentially identical at ∼0.1 mM. Reported values for kinetic properties of GYS1 (predominantly for the rabbit muscle preparation) are highly variable as noted by Roach and Larner [26], depending on the quality of the preparation and assay conditions thus it is difficult to find a definitive reference. Regardless, the two forms assayed here exist at the extremes of phosphorylation state (either stoichiometric or none) are not representative of physiological states *in vivo*, but do compare favourably with values reported for recombinant GYS1 purified from HEK293 cells which is also heavily phosphorylated and essentially inactive in the absence of G6P [17]. These results demonstrate that the GYS1:GN1 complex as prepared from insect cells is functional and exhibits properties similar to protein preparations from mammalian expression systems.

## Conclusions

Although it has long been proposed that glycogen synthase and glycogenin interact and this interaction plays a key role in glycogen biosynthesis in muscle and liver, to our knowledge no one has reported the preparation of a stoichiometric glycogen synthase:glycogenin complex in large quantities. Taking advantage of a bicistronic expression vector, we have set up conditions that allow successful and reproducible production of functional human GYS1:GN1 complex in insect cells. A large quantity of highly-pure stoichiometric GYS1:GN1 complex will be an invaluable tool to reveal its structural and biochemical properties for future studies.

## Contribution statement

E.Z., K.S., F.S. and R.W.H. designed the study. E.Z. optimised the protocol and produced and purified GYS1:GN1 complex. R.W.H. carried out biochemical and functional experiments and N.M. performed mass spectrometry analysis. F.S. supervised E.Z. R.W.H., KS, E.Z. and N.M. wrote the manuscript.

## Disclosure statement

The authors declare no conflict of interest associated with this study.

## Figures and Tables

**Table 1:** Phosphorylation sites on GYS1 identified by LC-MS.

Site	Classical nomenclature	Sequence	m/z [Da]
T54		^53^VtGDEWGDNYFLVGPYTEQGVR^74^	861.39

T372		^372^tNNFNVETLK^381^	630.29

S412		^403^KLYESLLVGsLPDMNK^418^	629.66

S486		^475^VIFHPEFLSSTsPLLPVDYEEFVR^798^	1451.23

S641	3a	^636^YPRPAsVPPsPSLSR^650^ ^636^YPRPAsVPPSPSLSR^650^ ^636^YPRPAsVPPsPSLSRHssPHQsEDEEDPR^664^ ^636^YPRPAsVPPsPsLSRHssPHQsEDEEDPR^664^ ^651^HSsPHQsEDEEDPR^664^	885.91 845.93 1214.47 1241.13 905.33

S645	3b		

S647			

S652	

S653	4

S657	5

S710	1b	^708^RNsVDTATSSSLSTPSEPLSPTSSLGEER^736^	1024.82
		^708^RNsVDTATSSSLSTPSEPLSPTSSLGEERN^737^	1062.83

S727		^709^NSVDTATSSSLSTPSEPLsPTSSLGEER^736^	1458.68
		^709^NSVDTATSSSLSTPSEPLsPTSSLGEERN^737^	1010.8

**Table 2:** Kinetic properties of phospho-(GYSb) and dephospho-GYS1 (GYSa).

Parameter	GYSb	GYSa
Specific activity (U/mg)^a^	16.7 ± 0.3^b^	17.1 ± 0.9^c^
*K*_m_(UDPG) (mM)	0.096 ± 0.001^d^	0.118 ± 0.004
*h*(UDPG)	1.34 ± 0.03	1.37 ± 0.05
Ka(G6P) (mM)	0.311 ± 0.005^c^	ND
*h*(G6P)	1.53 ± 0.02	ND
Phosphate content^e^	6.05 ± 0.23	Negligible

a Per mg GYS1.

b Determined using 4 mM UDP-glucose and 10 mM G6P.

c Determined using 4 mM UDP-glucose.

d Determined using 10 mM G6P.

e Mol phosphate per mol total protein (GYS1 + GN1).

## Footnotes

3 *Abbreviations used:* UDP, uridine diphosphate; G6P, glucose-6-phosphate; GSK3, glycogen synthase kinase-3; PKA, cAMP-dependent protein kinase/protein kinase A; CK, casein kinase; PP1, protein phosphatase-1.
